# ﻿New genera and new species of Catenulaceae (Bacillariophyta) from Coral Reef habitat of two Indonesia islands—Bawean and Sulawesi—A morphological approach

**DOI:** 10.3897/phytokeys.248.131839

**Published:** 2024-11-01

**Authors:** Oktiyas Muzaky Luthfi, Sulastri Arsad, Adrian Kryk, Yenny Risjani, Yunianta, Mateusz Rybak, Łukasz Peszek, Rafał J. Wróbel, Janice L. Pappas, Małgorzata Bąk, Andrzej Witkowski

**Affiliations:** 1 Institute of Marine and Environmental Sciences, University of Szczecin, ul. Mickiewicza 16, 70-383 Szczecin, Poland; 2 Faculty of Fisheries and Marine Sciences, Universitas Brawijaya, Jl. Veteran, Malang, 65145, Indonesia; 3 Center for Algae and Environment, (ALGAEN) LPPM, Universitas Brawijaya, Jl. Veteran, Malang, 65145, Indonesia; 4 Faculty of Agricultural Technology, Universitas Brawijaya, Jl. Veteran, Malang, 65145, Indonesia; 5 College of Natural Sciences, University of Rzeszów, ul. Zelwerowicza 4, 35-601, Rzeszów, Poland; 6 Department of Agroecology and Forest Utilization, University of Rzeszów, ul. Ćwiklińskiej 1A, 35-601 Rzeszów, Poland; 7 Engineering of Catalytic and Sorbent Materials Department, Faculty of Chemical Technology and Engineering, West Pomeranian University of Technology in Szczecin, Szczecin, Poland; 8 Department of Mathematics, University of Michigan, Ann Arbor, MI, USA; † Deceased

**Keywords:** Biodiversity hotspot, coastal habitats, diatom, epipsammic, taxonomy

## Abstract

Indonesia is listed as a region with high marine biodiversity, especially when considering the three large tropical ecosystems: seagrass beds, mangroves and coral reefs. It is likely that the biodiversity of diatoms in this area is also high. Samples for this research were taken from the remote area of Bawean Island and Tomini Gulf in Central Sulawesi. In this research, we explored marine benthic diatoms from coral reef areas and presented two new genera: *Paracatenula* and *Wallaceago*, and seven new species: *Paracatenulaporostriata***sp. nov.**, *Wallaceagoporostriatus***sp. nov.**, *Catenulaboyanensis***sp. nov.**, *C.komodensis***sp. nov.**, *C.decusa***sp. nov.**, *C.densestriata***sp. nov.**, and *Catenulopsisbaweana***sp. nov.** The new genus *Paracatenula* is characterized by its perforated cingulum, and the genus *Wallaceago* is distinguished by its proximal and apical raphe ends bent to the ventral side and the presence of a stauros in the mantle.

## ﻿Introduction

The family Catenulaceae initially had been described by Mereschkowsky (1902) who named one species, *Catenulapelagica*, and then transferred *Naviculaadhaerens* Mereschkowsky into *Catenula* (Silva 1980; Sundbäck and Medlin 1986). The Catenulaceae name came from the Latin word *catēna* and *ula* which literally has the meaning of a diminutive chain. The early described Catenulaceae was characterized by the number, form and arrangement of chloroplasts instead of valve characteristics. Since *Catenula* as the genus had been described at that time as having a single chromatophore, it was placed in the Monoplacatea (Sundbäck and Medlin 1986; Cox 1987). The recent family description is based on frustule characteristics like dorsiventral valves with the presence of two raphe slits. Additionally, features that are important to determine the genus are the number of areolae per striae, number of girdle bands, and the presence/absence of pores on the valve. Based on those criteria, the members of the diatom family Catenulaceae include several genera, e.g., *Amphora* Ehrenberg ex Kützing, *Catenula* Mereschkowsky, *Undatella* Paddock & Sims, *Halamphora* (Cleve) Levkov, *Oxyamphora* Stepanek & Kociolek, *Clevamphora* Mereschkowsky and *Catenulopsis* Kryk, Witkowski, Kociolek & Mayama (Paddock 1980; Round et al. 1990; Levkov 2009; Cox 2015; Stepanek and Kociolek 2016; Kryk et al. 2021). Based on a morphological approach, *Undatella* has a central keel raphe and a stauros similar to *Staurotropis* so this genus falls into the Bacillariales (Medlin and Kaczmarska 2004). A revised position of *Undatella* indicates that it belongs in the Thalassiophysales (Ruck and Theriot 2011; Louvrou and Economou-Amilli 2012). Another study based on a single molecular gene, SSU rDNA, resulted in this genus being moved back into Bacillariales (Ruck and Theriot 2011) and was stated as *incertae sedis* by www.marinespecies.org and Ashworth et al. (2017) (www.algaebase.org). The most recent classification is that *Undatella* belongs to Catenulaceae (www.diatombase.org, www.marinespecies.org). The genus *Halamphora* (Levkov 2009) previously was placed in Catenulaceae (Olivares-Rubio et al. 2017), but recently has been moved into the Family Amphipleuraceae (www.marinespecies.org, www.algaebase.org, www.diatombase.org). The genus *Clevamphora* was placed into the Subclass Bacillariophyceae*incertae sedis* (www.marinespecies.org, www.algaebase.org, www.diatombase.org). Based on a recent taxonomy review, the Catenulaceae family members are *Amphora*, *Catenula*, *Undatella*, *Oxyamphora* and *Catenulopsis* (www.diatombase.org, Kryk et al. 2021).

The genus *Catenula* has been described by Mereschkowsky (1902) as raphid diatoms which form ribbon-like colonies. *Catenula* is distinguished by valve asymmetry to the apical axis, eccentric raphe, clear helictoglossae (terminal nodule), and short terminal fissures on valve apices (Round et al. 1990; Kryk et al. 2021). The genus *Catenula* is very rarely reported in scientific publications to date, and only 6 species have been noted from all over the world. Since 1901, the species, *C.adhaerens*, had been described by Mereschkowsky, who two years later, published *C.pelagica*. Almost 100 years later, Witkowski et al. (2000) reported a new species as *C.robusta*, then another three species were described two decades later, i.e., *C.exigua* (Robert et al. 2019), *C.brotasiae*, and *C.javanica* (Kryk et al. 2021).

The monotypic genus *Catenulopsis* with its only representative, *Catenulopsiscatenulafalsa* Kryk & Witkowski, is characterized by dorsiventral valves with rectangular apices that are dull or obtusely formed. The position of the raphe is eccentric on the ventral side which has distal and proximal end bending on the ventral side. While the copulae have several pores (areolae), they have a punctate and lineolate form. Furthermore, the striation only can be found in the central margin (Kryk et al. 2021). This study presents the description of coral reef diatoms from the family Catenulaceae and describes new species of *Paracatenula*, *Wallaceago*, *Catenula* and *Catenulopsis*.

## ﻿Materials and methods

### ﻿Samplings

**Figure 1.**
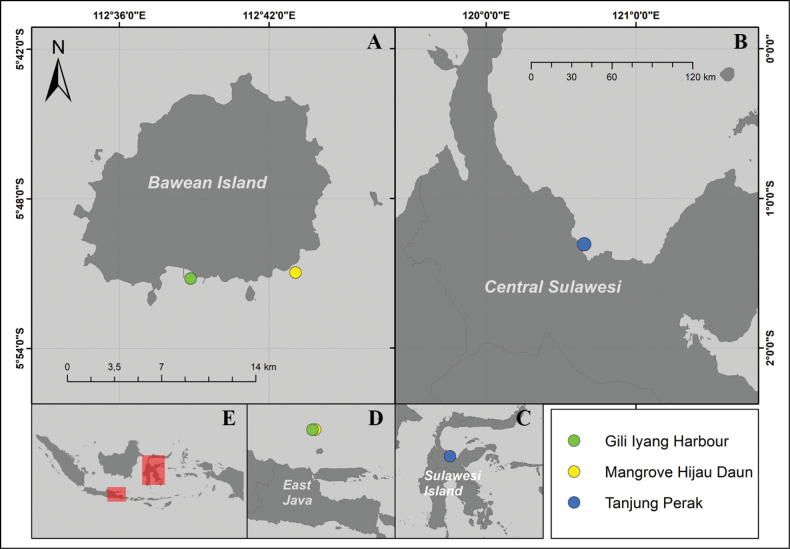
Map of sampling sites **A** sites Gili Iyang Harbour and MHD on Bawean Island **B** site Tanjung Perak on Central Sulawesi **C, D** detailed location of sampling areas on Sulawesi Island and East Java **E** location of Sulawesi and Java Island on Indonesian archipelago.

Sample materials were collected on 7 January 2021 at two sites on Bawean Island, Java Sea, Indonesia with local names *Mangrove Hijau Daun* – MHD (5°50'57.5"S, 112°43'3.6"E) and Gili Iyang (5°51'11.70"S, 112°38'51.10"E) (Fig. 1A). The diatom samples were taken from reef sand and coral rubble (pieces of dead coral) within the reef flat area which is located 1 km away from the mangrove swamp area or near the reef crest in MHD and around 500 m from the coast in Gili Iyang (Fig. 1D). The diatom samples from Sulawesi were taken from the intertidal area at Tanjung Perak of Tomini Gulf (1°18'2.974'′S, 120°37'37.009'′E) on 29 September 2022. They were collected from rocks in the coral reef area. The sampling point is part of Tomini Gulf, the largest gulf in Indonesia, which has 1,350 km of coastline, and the distance between the indentation mouth shorelines was 44.4 km (Fig. 1B).

### Light and scanning electron microscopy

The samples were treated with 10% HCl, removing carbonate minerals (ca. two days), and washed with deionized water thereafter. Samples then were treated by adding 37% H_2_O_2_ and boiling for 3–5 hours to remove organic matter (Górecka et al. 2021; Kryk et al. 2021). Finally, the boiled samples were rinsed five times with deionized water. Thereafter, the water suspension was pipetted onto coverslips, left at room temperature for 24 h until evaporation of the water, and mounted onto glass slides using Naphrax®. Light microscopy (LM) images were taken with a Zeiss Axio Scope A1 light microscope (Carl Zeiss, Jena, Germany), with a 100 × Plan Apochromatic oil immersion objective (n.a. = 1.40) equipped with a Canon EOS 500D camera (Li et al. 2015). For identification and terminology reference of diatoms, sources such as Witkowski et al. (2000) and Round et al. (1990) were used.

In addition, for scanning electron microscopy (SEM) imaging, a few drops of cleaned or raw material were placed on a Whatman (pore size 5 or 1 μm) Nuclepore® filter and rinsed twice with distilled water. Filters were mounted onto aluminum stubs and air-dried before coating with gold-palladium alloy using a sputter coater. SEM examination involved a Hitachi SU8020 from West Pomerania University of Technology in Szczecin (Poland), at the Faculty of Chemical Technology and Engineering, and a Hitachi SU8010 at the University of Rzeszów, Poland.

## ﻿Results

**Phylum: Bacillariophyta Karsten**

**Subphyllum: Bacillariophytina Medlin & Kaczmarska**

**Class: Bacillariophyceae Haeckel**

**Sub-class: Bacillariophycidae D.G. Mann**

**Order: Thalassiophysales D.G. Mann**

**Family: Catenulaceae Mereschkowsky**

### ﻿ Paracatenula

Taxon classificationPlantaeThalassiophysalesCatenulaceae

Witkowski, Luthfi & M.Rybak gen. nov.

ECD5BC3A-4635-5451-BCFD-D1C09D0752B2

#### Etymology.

The name of the genus is derived from its resemblance to *Catenula*. “Para” in Greek means alongside, besides, near, resembling, beyond, apart from, and abnormal, referring to the superficial similarity of the new genus to *Catenula* but does not conform to the type of the genus as typified with *C.pelagica* Mereschkowsky. *Paracatenula* means resembling *Catenula*.

#### Description.

Frustules strongly dorsiventral attached with valve faces to form short chains, plastid unknown. In girdle view, several bands perforated with one or two rows of small pores. Valves asymmetrical about the apical axis with dorsal margin gently arched and ventral margin straight usually with apices slightly deflected towards the ventral side. Raphe sternum positioned close to the ventral margin, raphe slits externally almost straight with simple proximal and apical ends. Striae on valve face usually absent or observed as shallow transapical grooves filled in with silica. On the dorsal mantle, solitary pore-like areolae present, whereas on the dorsal mantle, short striae usually composed of several small poroids. Valve face internally hyaline without any areolae, and valve mantle having a row of areolae along the valve margin, which is occluded by hymens. Internally, raphe slits are bent toward the dorsal margin and proximally terminate in a simple somewhat raised end, whereas apically, they terminate in oblique helictoglossae. Present sulcus in apical area. The sulcus clearly found in the internal view of the diatom valve after the distal ending (Fig. 3B).

### ﻿ Paracatenula porostriata

Taxon classificationPlantaeThalassiophysalesCatenulaceae

Luthfi, Witkowski & M.Rybak sp. nov.

9E3550DA-A039-584C-98B9-4144743D1B22

#### Type materials.

***Holotype***: Slide number SZCZ 27552 at repository of University of Szczecin.

***Isotype***: SZCZ 27553 at repository of University of Szczecin.

#### Type locality.

Rubble of coral reef at Gili Iyang harbour, Bawean Island, East Java, Indonesia

#### Etymology.

The species epithet ‘porostriata’ is a combination of the Latin words *porus* meaning pore or punctum and *striatus* meaning striated or having striations to show that this species consists of porous striations on the mantle and cingulum.

#### Distribution.

The diatom species *P.porostriata* sp. nov. has been consistently found in coral rubble specimens from both Gili Iyang harbor and MHD, Bawean Island.

#### Description.

**Figure 2.**
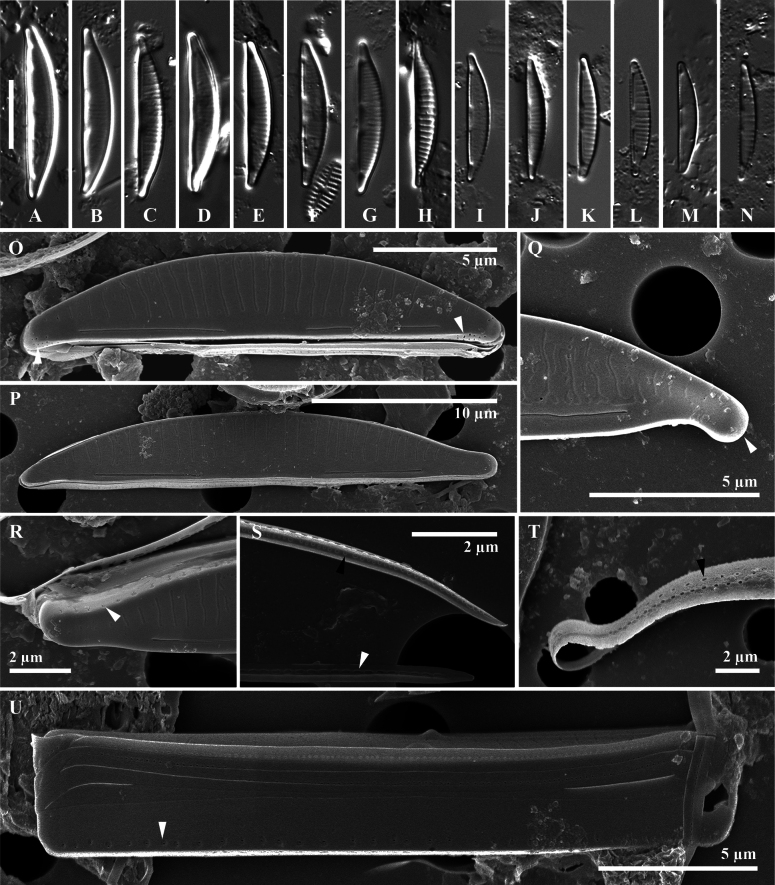
*Paracatenulaporostriata* Luthfi, Witkowski & M.Rybak, sp. nov. **A–N** light micrographs **O–U**SEM images of external valve view **O, P** external view of valves, unoccluded pores at one or both apices are present (white arrowhead, **O**) **Q** protracted apex of valve **R** pores on the valve mantle shown in the white arrowhead **S, T** cingulum perforated by biseriate striae externally at the dorsal side (black arrowheads) and one row of elongated pores internally at the ventral side (white arrowhead, **S**) **U** whole girdle view of frustule, with depressed areolae on valve mantle. Scale bar: 10 µm (**A–N**).

***Light microscopy*** (Fig. 2A–N): The valves are semi-lanceolate, apices broadly rounded, protracted and rostrate, which tend to deflect to the ventral side. The ventral margin is straight, and the dorsal side is smoothly arched or curved. Raphe slits are observed on the valve face as short lines. Valve length 10.1–25.4 µm (n = 25) and width 1.7–4.7 µm (n = 25). Striation is parallel in the middle and then slightly radial near the ends.

**Figure 3.**
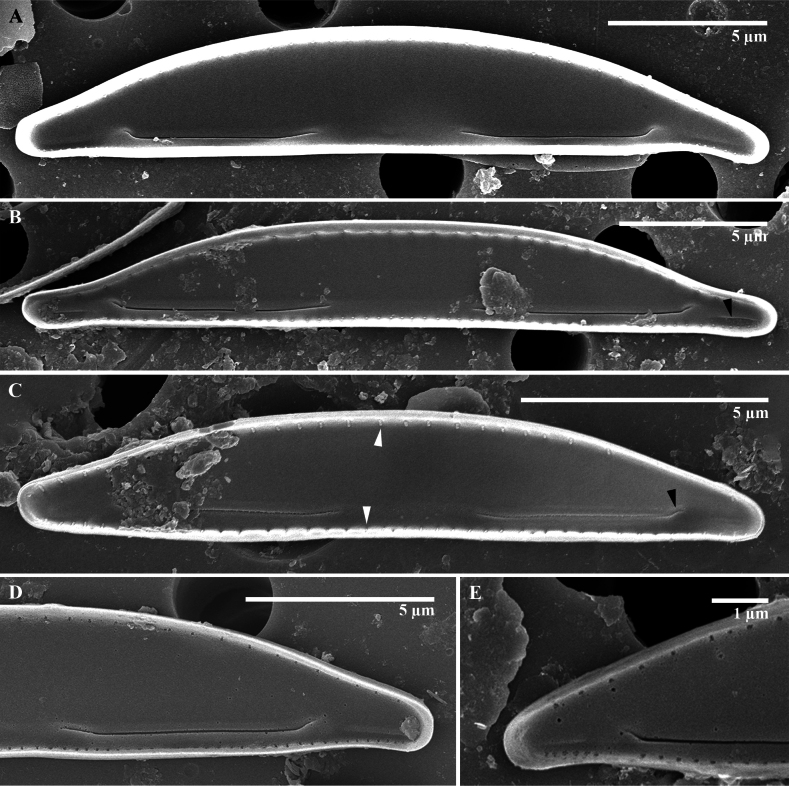
*Paracatenulaporostriata* Luthfi, Witkowski & M.Rybak, sp. nov. SEM images of internal view **A** valve with protracted apices **B** valve with linear grooves (Latin *sulcus*, plural *sulci*) on apices area (black arrowhead, also on **A–E**) **C** valve view showing occluded areola on ventral and dorsal valve mantel **D, E** internal apical raphe end bent and ending with indistinct helictoglossa.

***Scanning electron microscopy***: (Fig. 2O–U external view; Fig. 3A–E internal view): valve shape semi-circular to semi-lanceolate dorsiventral. The surface of the valve, both externally and internally, is completely flat. The transition between the valve face and valve mantle is sharp with a slightly diminished rib. Central area is distinct only on the valve face; lack of fascia on valve mantle. Axial area wide on the dorsal side and narrower on the ventral side (Fig. 2O, P). Externally, the raphe is lateral, short, simple and positioned in close proximity to the edge of the ventral valve face. Proximal raphe ends straight, distance to each other is 3.8 µm (n = 12). Distal raphe ends straight, distance to the apices is 2.6 µm (n = 11). Externally, transapical striae are arranged by one row along the dorsal face about 14–20 in 10 µm. Uniseriate striae observed along the valve face (Fig. 2O–R). Sometimes, scattered unoccluded pores are found on apices (Fig. 2O). Girdle band open with two rows of pores (Fig. 2S–T). Frustules contain five girdle bands and have a rectangular form in girdle view (Fig. 2U). Internally, the raphe slits are short, simple, and arcuate, with proximal and distal ends bent toward the dorsal side. Raphe fissure terminating in distinct helictoglossae (Fig. 3A–E). Line-like and depressed siliceous areolae (Latin *sulcus*, plural *sulci*) are present internally at the apical areas, approximately 1.3 µm in length (Fig. 3B).

### ﻿ Wallaceago

Taxon classificationPlantaeThalassiophysalesCatenulaceae

Witkowski, Arsad, Luthfi & M.Rybak gen. nov.

6DB00F7C-ED5D-5A8D-BDCB-E9BBFA11D2A1

#### Etymology.

The name of the genus is dedicated to Alfred Russel Wallace in recognition of his contribution to the biogeography of the Indonesian Islands. The ending “-ago” is used to denote an explorer or traveller.

#### Description.

Frustules strongly dorsiventral, plastid unknown, and girdle not observed. Valves asymmetrical about the apical axis. Raphe sternum close to the ventral side along the apical axis. Raphe slit straight with externally simple proximal and apical ends that bent towards the ventral margin. Transapical striae absent in the valve face, but short striae present on the ventral margin, which is composed of a series of small areolae. On the ventral side, a distinct, rhomboidal central nodule is observed. Valve face internally flat, central nodule with distinct siliceous deposition. Areolae occluded by hymenate structures. Raphe slits internal, exhibiting slight dorsal curvature; proximally elevated above central nodule deposition, terminating apically in indistinct helictoglossae.

### ﻿ Wallaceago porostriatus

Taxon classificationPlantaeThalassiophysalesCatenulaceae

Arsad, Witkowski, Luthfi & M.Rybak sp. nov.

4BF7ED44-23EB-5B1A-8E61-BC9899AB0480

#### Holotype.

Slide number SZCZ 28814 at the repository of the University of Szczecin.

#### Type locality.

Rock scrape in Tanjung Perak, Poso Pesisir Regency, Central Sulawesi/ Celebes, Tomini Gulf, Indonesia

#### Etymology.

This species is dedicated to Alfred Russel Wallace in gratitude for his contribution to exploring the Indonesian archipelago. The term *porostriatus* is a Latin adjective that means having porous striations or with striations composed of pores.

#### Distribution.

The new species so far is only found in Tanjung Perak, Sulawesi, Indonesia.

#### Description.

**Figure 4.**
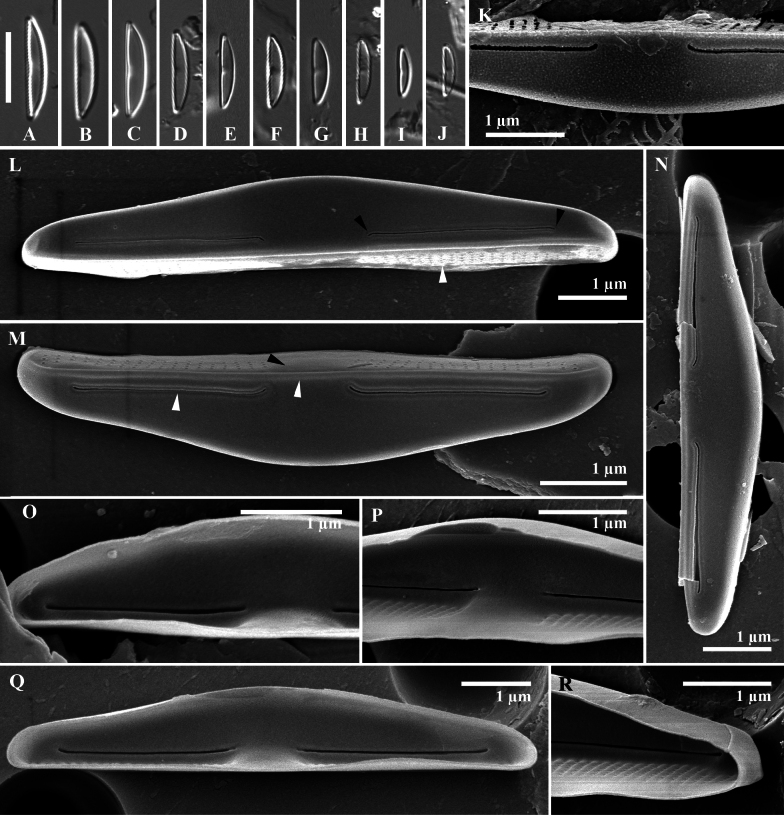
*Wallaceagoporostriatus* Arsad, Witkowski, Luthfi & M.Rybak, sp. nov. (**A–R**) **A–J** light microscopy images **K–R**SEM images **K–N** external valve view **O–R** internal valve view **L, M** striation in mantle consists of 2–5 tiny areolae (white arrowhead, **L**), proximal and distal raphe ends bent in the same direction towards the ventral side (black arrowheads, **L**) **M** a presence of stauros (black arrowhead), ornamented raphe and the edge of the valve by a rabbet (white arrowheads) **O–Q** raphe straight with simple ends **R** distinct virgae on the mantle with unoccluded pores. Scale bar: 10 µm (**A–J**).

***Light microscopy*** (Fig. 4A–J): The valves are dorsiventral, apices dull without protracted ends. The narrower valves are weakly protracted. Valve length 7.1–14.8 µm (n = 17) and width 1.6–3.1 µm (n = 17). Raphe branches cannot be found. The central area is resolvable with LM, marked by a light color in the middle of the ventral margin.

***Scanning electron microscopy*** (Fig. 4K–R): Valves exhibit semi-lanceolate dorsiventral morphology, transitioning to pyramidal form. The valve face presents as flat and smooth, devoid of striae ornamentation both externally and internally (Fig. 4K–R). The dorsal mantle transitions gradually from the valve face, while the ventral mantle transition is abrupt, characterized by a distinct groove resembling a rabbet at the valve face edge (Fig. 4L, M). Ventral mantle seriation is uniseriate, with 70–80 areolae per 10 µm. Areolae are rounded and separated by a pore-free, hyaline silica thickening, forming a stauros (Fig. 4M). Seriation grooves are prominently marked internally (Fig. 4P–R). The dorsal mantle lacks areolae (Fig. 4P, R). The raphe externally filiform with a simple, rabbet-like profile, positioned close to the ventral valve margin (Fig. 4L–N). External proximal raphe ends simply, slightly deflected ventrally, and somewhat distant from each other (1.1 µm). Distal raphe endings are simple, lacking fissures, and bent towards the ventral margin. Internally, raphe endings straight, terminating slightly towards the dorsal valve at the central area (Fig. 4O, P). Distal raphe endings are straight and situated close to the apices (0.5 µm), with indistinct or absent helictoglossae (Fig. 4Q, R). Girdle bands were not observed in the specimens examined.

### ﻿ Catenula boyanensis

Taxon classificationPlantaeThalassiophysalesCatenulaceae

Luthfi, Witkowski & M.Rybak sp. nov.

7A08A0FE-0811-5D12-B04A-2A25ABD89C4C

#### Holotype.

Slide number SZCZ 27552 at the repository of the University of Szczecin.

#### Type locality.

Sand and coral boulders of coral reef at Gili Iyang harbour Bawean Island, East Java, Indonesia.

#### Etymology.

The species name is derived from local Bawean Island people called Boyan.

#### Distribution.

The diatom species *C.boyanensis* has been regularly observed in samples from the harbour of Gili Iyang on Bawean Island.

#### Description.

**Figure 5.**
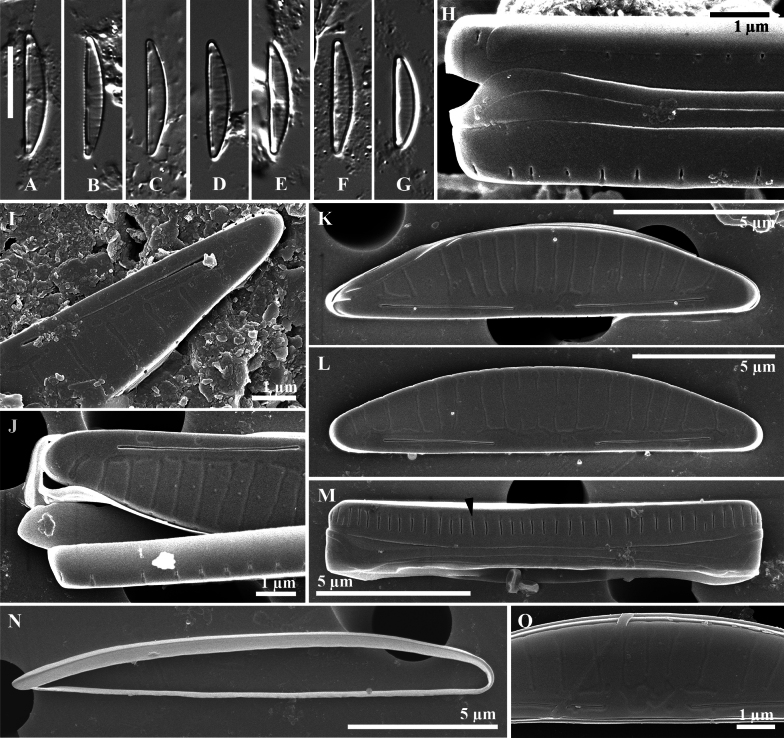
*Catenulaboyanensis* Luthfi, Witkowski & M.Rybak, sp. nov. **A–G** light microscopy images **H–O**SEM images in external view **H, M** girdle view of frustule showing rectangular with obtuse shape on corners and a row of elongated striae on mantle (**M**, black arrowhead) **I** detail of raphe branch **J** detail of apical part of a frustule with girdle band **M** the mantle of valve **K–L** external valve view showing transapical irregular grooves and the central area **N** open unperforated cingulum **O** detail of frustule central part. Scale bar: 10 µm (**A–G**).

***Light microscopy*** (Fig. 5A–G): The valves are semi-lanceolate, dorsiventral. Raphe is clearly observed through the ventral area with proximal raphe endings distant from each other and distal raphe endings distant to apices (Fig. 5A–F). Valve length 8.2–22.2 µm, n = 29 and width 2.1–4.9 µm, n = 17. Transapical striae can be observed on the dorsal face. Apices obtusely rounded with distinct helictoglossae.

**Figure 6.**
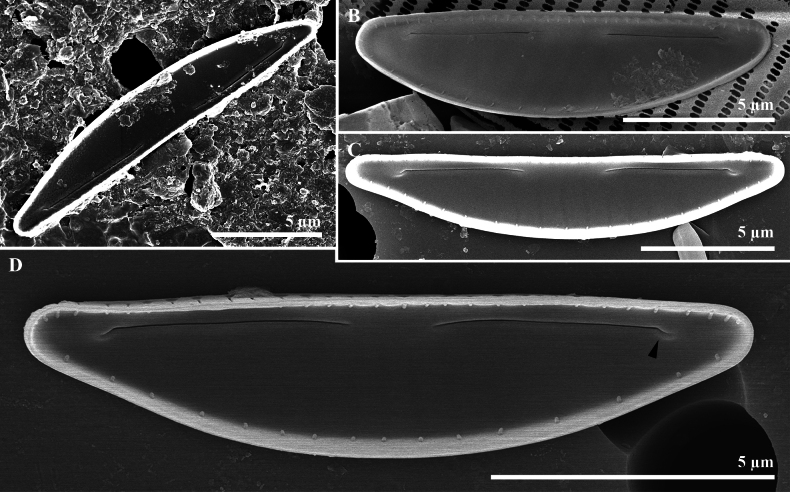
Internal view of *Catenulaboyanensis* Luthfi, Witkowski & M.Rybak, sp. nov. SEM images **A–D** internal valve view showing arcuate raphe slit, a row of linear areolae on mantle **D** detail of raphe branch and proximal raphe endings with apical raphe ending bent and finishing with small helictoglossae (arrowhead).

***Scanning electron microscopy*** (Figs 5, 6): Frustules exhibit significant dorsiventral. Several non-perforated girdle bands compose the frustule (Fig. 5H, M). Valves display strong asymmetry around the apical axis, with a gently arched dorsal margin and straight ventral margin (Fig. 5K, L). The raphe sternum is positioned near the ventral margin, with nearly straight raphe slits (Fig. 5L). Raphe occupies a more central location rather than along the ventral margin, with proximal raphe endings distantly spaced (1.5–3.8 µm) (Fig. 5K, L). External proximal and apical ends are filiform, short, and straight (Fig. 5I). Transapical striae appear as shallow grooves on the valve face, with a row of striae visible on the dorsal mantle (Fig. 5H, J, M). The internal valve face is flat. Small areolae on the valve surface are covered by a thin hymen layer. Internally, raphe slits bend very slightly toward the dorsal margin, maintain straight proximal ends, and terminate apically in helictoglossae (Fig. 6D).

### ﻿ Catenula decusa

Taxon classificationPlantaeThalassiophysalesCatenulaceae

Luthfi, Witkowski, Arsad & M.Rybak sp. nov.

CFBE38DD-CAA3-5E3A-B876-7336F0CC58A5

#### Type materials.

***Holotype***: Slide number SZCZ 27552 at repository of University of Szczecin.

**Figure 7.**
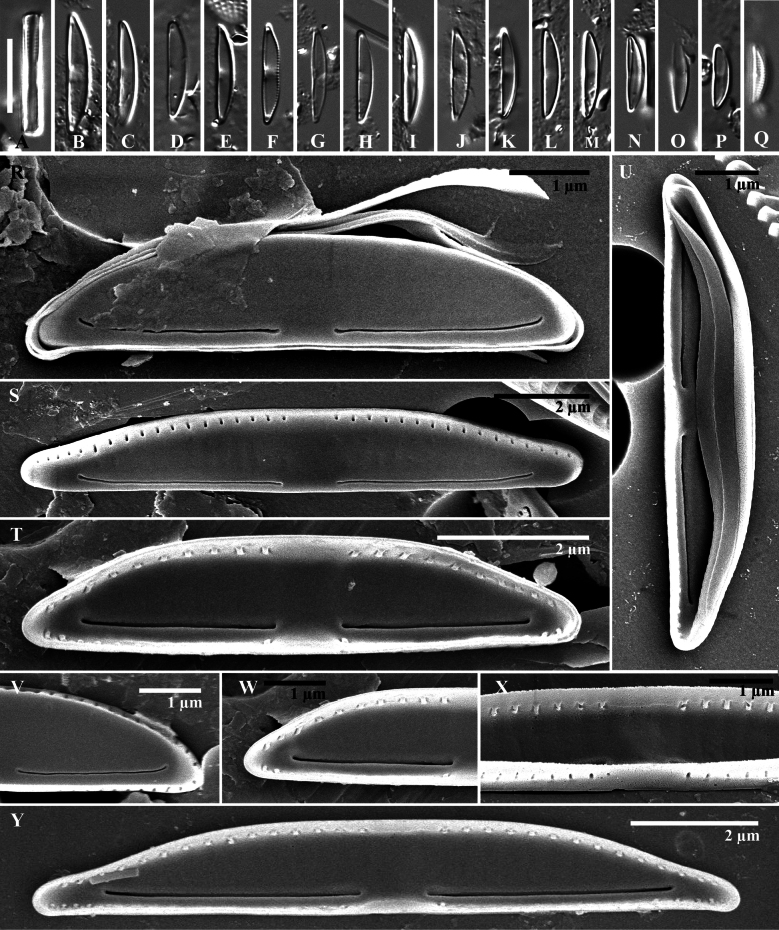
*Catenuladecusa* Luthfi, Witkowski, Arsad & M.Rybak, sp. nov. **A–Q** light micrographs **R–Y**SEM images **R, S, V** external view **T, U, W–Y** internal view **R** external view of frustule with three cingula **S** external valve view with rectangular central area created by striation interruption in dorsal and ventral margin **T, Y** internal view of entire valve **U** valve with open unperforated girdle band **V** external view an arcuate raphe with simple proximal and apical end **W** straight raphe slit in internal valve view **X** rectangular central area with interrupted dorsal and ventral mantle striation. Scale bar: 10 µm (**A–Q**).

***Isotype***: Slide number SZCZ 28814 at repository of University of Szczecin (Fig. 7S).

#### Type locality.

Sand and coral boulder of coral reef at Gili Iyang harbour, Bawean Island, East Java, Indonesia.

#### Etymology.

The species name is derived from the Latin word *decus* which literally means an ornament, decoration, or embellishment. The new species exhibits a distinct central area.

#### Distribution.

The diatom species *C.decusa* has a unique distribution, being found exclusively in Bawean and Sulawesi Islands, Indonesia. Interestingly, both of these locations share a similar habitat, characterized by the presence of coral reef areas.

#### Description.

***Light microscopy*** (Fig. 7A–Q): The valves are semi-lunate or semi-lanceolate dorsiventrally. Thickening silica in the central area is very clear under LM. A row of linear areolae can be distinguished in the dorsal margin (Fig. 7F). Valve length 8.2–16.5 µm, n = 18 and width 1.9–2.9 µm, n = 17. Apices broadly rounded with indistinct, dot-like helictoglossae. The frustule is rectangular in girdle view, 1.1 µm depth. Raphe slits observed in ventral area.

***Scanning electron microscopy*** (Fig. 7R–Y): Frustules semi-circular to semi-lanceolate, dorsiventral; valve face flat, smooth, abruptly transitioning to mantle. Distinct rhomboidal central nodule observed on ventral and dorsal sides (Fig. 7S, T). Valve face devoid of transapical striae; striae present on dorsal and ventral mantle (Fig. 7R). Raphe sternum positioned near ventral margin; raphe slits straight medially, curving towards apices. External proximal raphe ends simple, slightly expanded; distal ends simple, close to apices (x–=0.72 μm, n = 21), deflected dorsally. Central nodules and helictoglossae indistinct. Dorsal mantle striation density 35–40 in 10 μm; ventral mantle 40–50 in 10 μm. Internal raphe filiform, straight (Fig. 7T, U, Y); mantle areolae occluded, flask-shaped (Fig. 7X). Girdle open, comprising multiple unperforated plain bands (Fig. 7R, U).

### ﻿ Catenula komodensis

Taxon classificationPlantaeThalassiophysalesCatenulaceae

Witkowski, Risjani, Yunianta, M.Rybak & Luthfi sp. nov.

8BC8B6D9-F6DA-5141-942D-0D1F749319EB

#### Type materials.

***Holotype***: Slide number SZCZ 27552 at repository of University of Szczecin.

**Figure 8.**
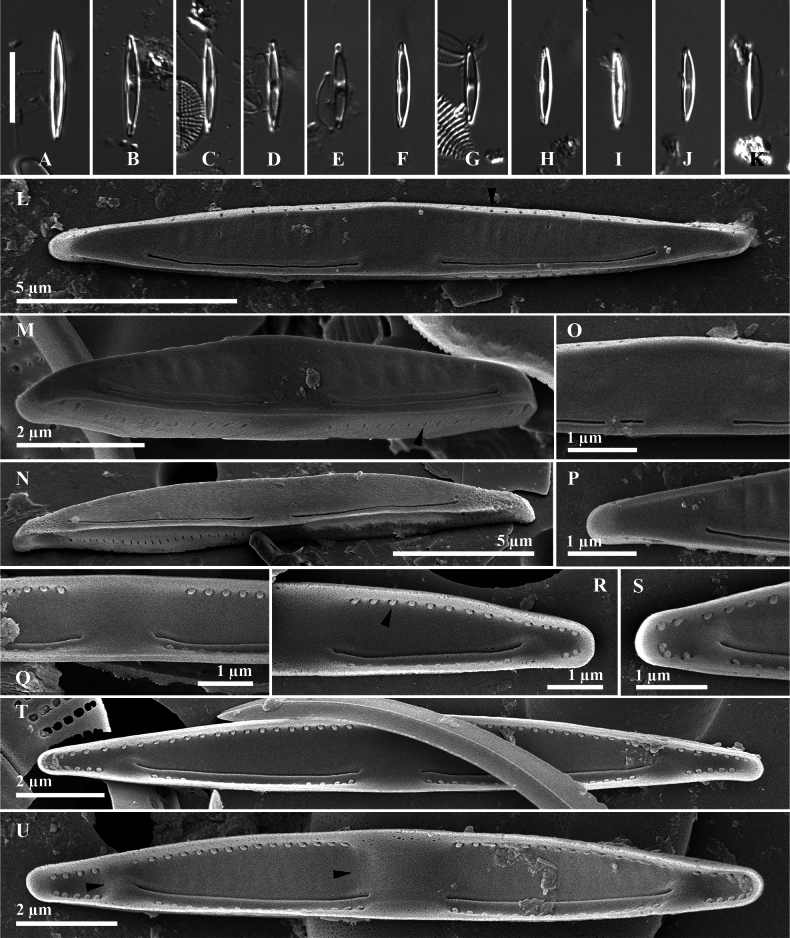
*Catenulakomodensis* Witkowski, Risjani, Yunianta, M.Rybak & Luthfi, sp. nov. **A–K** light micrographs **L–U**SEM images. **L–N** external view on entire frustules; note a row of slit-like ventral and dorsal mantles (arrowheads) **O** detail of proximal raphe ending, externally **P** detail of apical raphe ending, externally **Q–S** detail of internal proximal raphe ending, arcuate raphe slit and distal raphe ending, respectively; note a row of linear small protruding flaps (**R** arrowhead) **T–U** internal valve view with 2 thickenings of silica on central area and near slit ends (**U** arrowheads). Scale bar: 10 µm (**A–K**).

***Isotype***: Slide number SZCZ 28814 at repository of University of Szczecin (Fig. 8N).

#### Type locality.

Sand and coral boulders of coral reef at Gili Iyang harbour Bawean Island, East Java, Indonesia.

#### Etymology.

The species name is derived from the Komodo dragon that is endemic to 4 islands: Komodo, Rinca, Flores, and Gili Motang, East Nusa Tenggara, Indonesia.

#### Distribution.

The diatom species *C.komodensis* is distributed on Bawean and Sulawesi Islands, Indonesia.

#### Description.

***Light microscopy*** (Fig. 8A–K): The valves are lanceolate, dorsiventral to linear-lanceolate with straight to less convex margins. Apices are narrowly pointed. Thickened silica in the central area was observed clearly on some valves under LM. Valve length 8.3–15.6 µm, n = 13 and width 1.7–2.6 µm, n = 13.

***Scanning electron microscopy*** (Fig. 8L–U): Valve face flat, abruptly transitioning to mantle. Raphe filiform, positioned near ventral margin externally and internally (Fig. 8L–N, T–U). External raphe slits arcuate; proximal endings simple, dorsally oriented, distantly spaced (1.1–1.9 μm). Distal raphe endings simple, terminating 0.8–2.8 μm from apices. Areolae absent on dorsal and ventral valve surfaces. Dorsal side exhibits shallow or indistinct parallel grooves. Internal raphe slits curved; apical endings deflected dorsally (Fig. 8T–U). Internal distal raphe endings simple, without fissures, terminating 1.7–1.8 μm from apices. Helictoglossae indistinct (Fig. 8S). Dorsal and ventral sides striated; areolae occluded by dome-like structures. Striae density slightly lower dorsally; secondary silica deposition observed on ventral valve side (Fig. 8U). Striae density 34–35 in 10 μm ventrally, 35 in 10 μm dorsally (n = 13).

### ﻿ Catenula densestriata

Taxon classificationPlantaeThalassiophysalesCatenulaceae

, Luthfi, Witkowski, M.Rybak & Arsad sp. nov.

2ACF84F6-ECA3-5623-BD0B-A439C9C850A5

#### Holotype.

Slide number SZCZ 27553 at the repository of the University of Szczecin.

#### Type locality.

Sand and rubble of coral reef at Daun, Bawean Island, East Java, Indonesia.

#### Etymology.

The name of the species was derived from Latin meaning “densely packed.” This species has dense striation in the dorsal area.

#### Distribution.

The diatom species *C.densestriata* is found solely on Bawean Island and Tiga Warna Beach in East Java, Indonesia.

#### Description.

**Figure 9.**
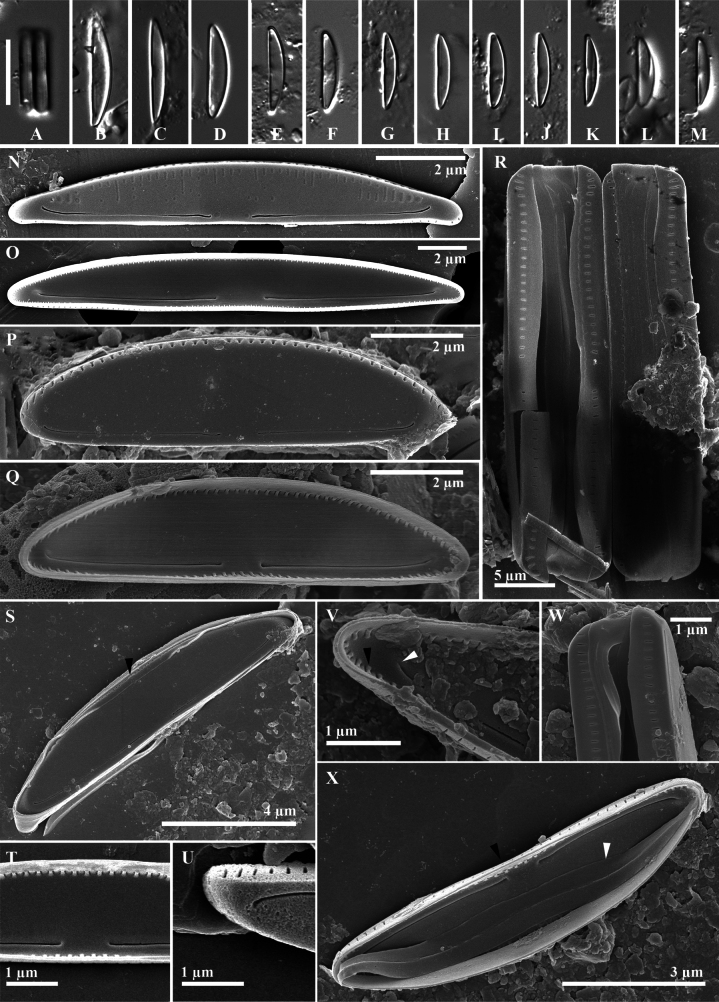
*Catenuladensestriata* Luthfi, Witkowski, M.Rybak & Arsad, sp. nov. **A–M** light micrographs **N–X**SEM images **N** external view of valve is semi-lanceolate dorsiventral and semi-circular (**P, S**) **O, Q, X** internal view of valves **R** two frustules are attached to each other by the valve face **S** areolae on dorsal mantle **T** a close-up of the detail of the proximal raphe ending as simple and straight **U** detail of the apical raphe ending deflected on dorsal side **V** detail of internal view, indistinct helictoglossa (white arrowhead) and linear occluded areolae (black arrowhead) **W** detail of frustule showing linear areola on mantle valve with several cingula **X** unperforated girdle band is shown at white arrowhead. Scale bar: 10 µm (**A–M**).

***Light microscopy*** (Fig. 9A–M): The frustules are rectangular in girdle view, 1.8 μm deep, joining each other on the valve face (Fig. 9A). Valves exhibit asymmetry and are dorsiventral, characterized by a curved dorsal margin and a straight ventral margin. Some dorsal margins are slightly flat in the middle, as semi-lanceolate (Fig. 9C, G, J, K, M). Valve apices cuneate with dot-like helictoglossae. Raphe slits positioned on the ventral side close to the margin (Fig. 9B–E, H). The terminal raphe ends are closed. Valve length 10.7–17.5 µm, n = 15 and width 1.8–3.5 µm, n = 14. The striae on the dorsal side are indistinct.

***Scanning electron microscopy*** (Fig. 9N–X): Valves semi-lanceolate, dorsiventral, with flat faces transitioning abruptly to dorsal and ventral mantles (Fig. 9N–Q). Short chains formed; plastid unknown. Externally, shallow fine radiate striae visible dorsally, absent ventrally (Fig. 9N). Linear pores present in rows on dorsal and ventral mantles (Fig. 9S, X). Internally, valve face lacks parallel striation; occluded pores as small protruding flaps on dorsal and ventral sides, density 40–50 in 10 µm dorsally, 40–55 in 10 µm ventrally. Raphe slits arcuate, deflected to same side. Internal raphe slightly terminated by indistinct helictoglossae toward dorsal side. Proximal raphe endings more distant internally (mean 1.5 µm, n = 14) than externally (mean 1.26 µm, n = 11). Central nodule indistinct. Girdle bands open, unperforated, comprising at least four cingula.

### ﻿ Catenulopsis baweana

Taxon classificationPlantaeThalassiophysalesCatenulaceae

Luthfi, Witkowski, M.Rybak & Kryk sp. nov.

0BAE1F58-8CBB-50C4-A7A7-1C377D7D404F

#### Holotype.

Slide number SZCZ 27552 at the repository of the University of Szczecin.

#### Type locality.

Sand and rubbles of coral reef at Gili Iyang harbour, Bawean Island, East Java, Indonesia.

#### Etymology.

The name of the species is derived from the geographic location of the type habitat, i.e., Bawean Island. The meaning of *Bawean* in Sanskrit is sunlight.

#### Distribution.

The diatom species *Ca.baweana* has a unique distribution, being found exclusively on Bawean Island, Indonesia.

#### Description.

**Figure 10.**
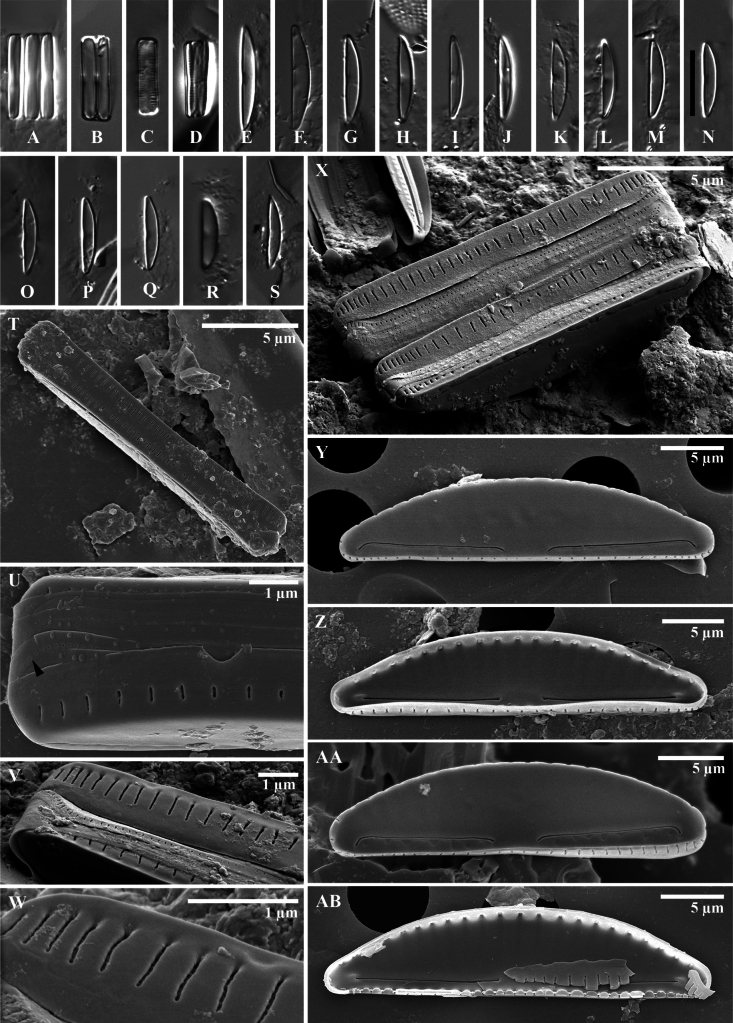
*Catenulopsisbaweana* Luthfi, Witkowski, M.Rybak & Kryk, sp. nov. **A–S** light micrographs **T–AB**SEM images **T** girdle view of the whole frustule **U** perforated valvocopula of frustule **V, W** detail of striation in the ventral mantle **X** frustule of diatoms attached at valve face **Y, AA** external view of valves semi-lanceolate dorsiventral with raphe branches. Proximal and apical endings deflected to ventral side **Z–AB** internal valve view shows simple raphe slits. Scale bar: 10 µm (**A–S**).

***Light microscopy*** (Fig. 10A–S): Valves are asymmetrical, dorsiventral, with the dorsal margin curved and the ventral margin straight. Valves are 10.7–16.1 μm long (n = 19) and 1.8–3.1 μm in width (n = 19). The apices look subcapitate when focusing on multiple planes using LM; the helictoglossae appear as a darker grey spot. A white line appearing near the ventral margin is a raphe. The frustule in girdle view is rectangular or widely rectangular because it consists of several cingula. Transapical striae are difficult to resolve with LM.

***Scanning electron microscopy*** (Fig. 10T–AB): Frustules strongly dorsiventral, rectangular in girdle view around 1.9–2.9 µm deep (Fig. 10T–V, X). The girdle is composed of several perforated bands (2–3 cingula), each with uniseriate small pores, 50–65 in 10 μm. Valves strongly asymmetrical about the apical axis, 8.8–17.1 μm long (n = 17) and 1.7–2.9 μm in width (n = 9). External and internal valves are flat and end abruptly at the mantle. The dorsal margin is gently arched, and the ventral margin is straight, apices obliquely cuneate. Raphe sternum is positioned close to the ventral margin, raphe slits almost straight. External proximal and apical ends are simple, bent towards the valve ventral margin. Proximal raphe ends somewhat distant from each other, 1.3–1.9 μm. Transapical striae present only on the valve face, dorsal and ventral mantle, and composed of long linear slits, 22–34 in 10 μm (n = 12). The internal valve face is flat. The areolae are closed with hymenate occlusions. Internally, raphe slits are very straight and skewed towards the dorsal area, and the proximal raphe ends terminate in a slightly expanded drop-like structure, whereas apically, they terminate in small helictoglossae.

## ﻿Discussion

### ﻿The new genera *Paracatenula* and *Wallaceago*

*Paracatenula* has unique features that make it recognisable, including its shape, striation in the mantle, girdle band, and a slit-like depression in the apical area, which we call a sulcus. The valve margin of the new genus has a dorsal convex and a ventral straight side, sharing similarities with *Catenula*. However, the valve apices in the new genus are protractedly deflected toward the ventral margin, which cannot be found in *Catenula*. The most similar genus which has the pointed valve ends that curve or bend downward is *Halamphora*, e.g., *Halamphoranormanii* (Rabenh) (Levkov 2009) and *H.montana* (Krasske) (Levkov 2009), which have capitate valve apices in shape. In contrast, *Paracatenula* has rostrate valve apices (Table 1).

**Table 1.** Morphological comparison of *Paracatenula* and *Wallaceago* with the most similar genera.

	*Paracatenula*	*Wallaceago*	*Catenulopsis*	*Catenula*	*Amphora*	*Lunella*	*Parlibellus*
	This study	This study	Kryk et al. (2021)	Mereschkowsky (1902)	Kützing (1844)	Snoeijs (1996)	Cox (1988)
Valves	strongly asymmetric and dorsiventral	strongly asymmetric, dorsiventral, semi–rhombic	strongly asymmetric and dorsiventral	strongly asymmetric and dorsiventral	asymmetric and dorsiventral	semi-lanceolate and dorsiventral	symmetric, bluntly lanceolate
Raphe position	eccentric, near ventral mantle	eccentric, near ventral mantle	eccentric, near ventral mantle	eccentric, near ventral mantle	moderate to strong eccentric toward ventral mantle or valve margin, raphe ledge present	near the ventral margin	on valve face, in the centre
Raphe shape	biarcuate	straight biarcuate	slightly biarcuate or sinusoidal	biarcuate	straight to biarcuate (sigmoid)	slightly biarcuate	straight
Raphe distal Endings	bent to dorsal, distant to poles	curved to ventral, distant to poles	curved to ventral, distant to poles	bent to dorsal, distant to poles	curved to dorsal face	terminating to ventral	terminating to secondary side
Raphe proximal Endings	straight, distant	close and curved to ventral	close and curved to ventral	straight, distant	terminate on ventral; on valve face; close to distant	straight to deflected on ventral; close	straight and distant
Sternum	narrow	none	narrow	narrow	narrow	narrow	narrow
Valve face/ mantle transition	abrupt	abrupt	abrupt	abrupt	abrupt	gradual both ventral and dorsal	gradual
Helictoglossae	well developed	poorly developed	poorly developed	well developed	poor to well-developed	well developed	well developed
Cingulum	open, perforated small pore	unknown	several plain porous copulae	open, non–porous band	plain or perforated, one or more areolae	porous copulae	wide girdle region, perforated

Striation in the valve mantle of *Paracatenula* is ornamented by single tiny uniseriate areolae. In other dorsiventral diatoms, such as *Catenula* and *Catenulopsis*, the mantle is featured by a row of elongated slits which are positioned perpendicularly to the surface of the valves (Fig. 2U). *Paracatenula* features an intricate unique cingulum structure with an open channelled and perforated band. Externally, on the dorsal side, the cingulum is ornamented with biseriate striae, creating a double row of fine, closely spaced lines. Internally, on the ventral side, it features a single row of elongated pores (Fig. 2S, T). The absence of such perforation patterns in *Catenula* highlights this distinctive morphological feature of *Paracatenula*. Several diatom genera with a perforated cingulum include *Catenulopsis* and *Halamphora*. Frustules of *Catenulopsis* are supported by several perforated girdle bands (Kryk et al. 2021). Another dorsiventral diatom which is composed of girdle bands perforated with a few rows of poroids is *Halamphoracatenulafalsa* (Witkowski et al. 2016). However, the two unique types of cingula striation previously described are only found in *Paracatenula*. The perforated cingulum is common and found in centric diatoms, e.g., *Thalassiosira* and *Stephanodiscus* (Round et al. 1990).

The genus *Paracatenula* exhibits several distinctive characteristics that justify its classification within the Catenulaceae family (Table 1). The frustules of *Paracatenula* are rectangular in girdle view, have asymmetric dorsiventral valves in the apical plane, the raphe position is eccentric close to the ventral margin, valve apices are protracted and rostrate, there is striation in the mantle, and there is a plain valve face with transapical ribbing (Round et al. 1990; Levkov 2009; Pliński and Witkowski 2020). Additionally, the family Catenulaceae exhibits a variety of apical valve morphologies. For instance, *Catenula* has no protracted apices, while *Amphora* and *Halamphora* do have protracted, capitate apices that terminate on the dorsal mantle. Moreover, the protracted, rostrate valve apices of *Paracatenula* are rarely reported from other genera of Catenulaceae. Only *C.pelagica* has been reported to have slightly rostrate apices (Round et al. 1990).

The newly described genus *Wallaceago* differs from all established catenuloid genera in terms of its valve shape, stauros, and raphe. *Wallaceago* exhibits a distinctive valve morphology characterized by a semi-rhombic, dorsiventral shape with broadly rounded apices. The valves display a pronounced bilateral flattening along the dorsiventral axis while maintaining a semi-rhombic outline tapering towards the apices. This combination of dorsiventral compression and rounded apical terminations distinguishes *Wallaceago* from other closely related genera, e.g., *Amphora*, *Halamphora*, *Catenula* and *Catenulopsis* which possess valves with a semi-lanceolate, dorsiventral form (Barber and Haworth 1981; Round et al. 1990; Robert et al. 2019; Stepanek and Patrick Kociolek 2019; Kryk et al. 2021). So far, only *Wallaceago* exhibits these features. Moreover, striation in the mantle of *Wallaceago* is characterized by a distinctive pattern on the ventral side (70–80 striae per 10 µm) which is interrupted by a stauros. The stauros has been previously reported in *C.adhaerens* from Zimbros, Brazil but it is located in the central area of the valve face (Garcia and Talgatti 2011, p. 103, figs 16, 17). Note, in our opinion those described specimens are similar to *C.decusa* sp. nov. in this paper. The stauros is a distinctive valve feature found primarily in *Catenula* (Garcia and Talgatti 2011) and in the freshwater diatom, genus *Stauroneis* (Levkov et al. 2016). For distinguishing the fascia from the stauros, a key morphological feature in certain diatom taxa, refer to the comprehensive review by Cox (2012).

*Wallaceago* exhibits a distinctive biarcuate raphe system, characterised by both proximal and distal ends bent towards the ventral side of the valve. A unique feature of the raphe in this genus is the ornamentation along the slit, which resembles a framed profile. While *Wallaceago* shares some similarities in raphe morphology with the genus *Catenulopsis*, notable differences exist. In *Catenulopsis*, the raphe displays a more pronounced sinusoidal curvature, whereas in *Wallaceago*, the raphe tends to be less curved, with a straighter trajectory (Kryk et al. 2021).

The genus *Wallaceago* shows several distinctive characteristics that justify its classification within the Catenulaceae family. The frustule of *Wallaceago* has asymmetric dorsiventral valves, is rectangular in girdle view, the raphe position is eccentric close to the ventral margin, valve apices are never protracted, and there is striation in the mantle with a plain valve face with transapical ribbing (Round et al. 1990; Levkov 2009; Pliński and Witkowski 2020). The genus *Wallaceago* appears similar to the genus *Catenulopsis* in terms of the raphe position and direction of the raphe ends. Another shared characteristic between *Wallaceago* and *Catenulopsis* is the presence of striations on the valve mantle. However, a notable difference lies in the specific arrangement and nature of these striations. In *Wallaceago*, the striations are confined solely to the ventral mantle and consist of uniseriate areolae. In contrast, *Catenulopsis* exhibits striation patterns on both the dorsal and ventral mantles, with these striations appearing as single rows of puncta or pores. In addition, *Catenulopsis* exhibits areolate striations composed of columnar or lanceolate shapes similar to *Amphora*.

### ﻿Novel *Paracatenula* species

*Paracatenulaporostriata* and *C.javanica*, exhibit intriguing similarities and differences. Both species possess a dorsiventral valve, a characteristic that is shared among catenuloid diatoms. Their transapical striae, a key feature in their structure, are barely resolvable when observed through the LM. However, a notable distinction lies in their apical forms. *P.porostriata* is characterized by protracted rostrate apices, a feature that is absent in *C.javanica*. As indicated in Table 2, there is a significant difference in the lengths of the two species. Specifically, the length of the newly described species, ranges from 10.1–26.6 µm. On the other hand, *C.javanica*, is shorter, with a length ranging from 9.7–15.4 µm (Table 2). Under LM observation, *P.porostriata* is easily misunderstood to be *C.javanica* (see Kryk et al. 2021; figs 53, 60) which has a dorsiventral shape with slightly protracted rostrate apices. Both species also have refractive proximal and distal raphe ends that appear as dots in a bright colour.

**Table 2.** Comparison of newly described species with the most similar taxa.

	*Paracatenulaporostriata* sp. nov.	*Wallaceagoporostriatus* sp. nov.	*Catenulaboyanensis* sp. nov.	*Catenuladecusa* sp. nov.	*Catenulakomodensis* sp. nov.	*Catenuladensestriata* sp. nov.	*Catenulabrotasiae*	*Catenulajavanica*
	This study	This study	This study	This study	This study	This study	Kryk et al. (2021)	Kryk et al. (2021)
Length (μm)*	10.1–26.6	5.2–8.6	8.2–22.2	6.8–16.5	8.1–19.3	8.2–20.3	8.5–11.8	9.7–15.4
Width (μm)*	1.7–4.7	0.9–1.2	1.1–4.9	1.0–1.7	1.1–2.1	1.6–3.5	2.8–3.5	2.4–3.5
Valve shape	semi-lanceolate dorsiventral	semi-rhombic dorsiventral	semi-lanceolate dorsiventral	semi-lanceolate dorsiventral	lanceolate dorsiventral	semi-lanceolate dorsiventral	dorsiventral	dorsiventral
Girdle shape	rectangular	rectangular	rectangular	rectangular	rectangular	rectangular	rectangular	rectangular
Sternum	narrow	narrow	absent	absent	narrow	absent	absent	narrow
Raphe slits path	straight	biarcuate	straight	arcuate	biarcuate	arcuate	straight	straight
Distal raphe ending	straight (external), deflected to dorsal (internal)	deflected to ventral (external), straight (internal)	straight (external), deflected to dorsal (internal)	deflected to dorsal (external), straight (internal)	straight (external), deflected to dorsal (internal)	deflected to dorsal (external), straight (internal)	straight (external), deflected to dorsal (internal)	straight (external), deflected to dorsal (internal)
Proximal raphe ending	straight, distant (external and internal)	deflected to ventral (external), straight (internal), close	straight, distant (external and internal)	straight, close (external and internal)	deflected to ventral, close (external and internal)	straight, close (external and internal)	straight, close (external and internal)	straight (external), deflected to dorsal (internal), distant
Raphe slits length (μm)	11.8	4.7	9.1	6.8	9	9.9	8.4	6.8
Raphe position	ventral area	ventral area	ventral area	ventral area	ventral area	ventral area	ventral area	ventral area margin
Central nodule	absent	absent	absent	absent	absent	absent	present	absent
Central area	present	present	present	present	present	present	present	present
Apices	broadly rounded, protracted, rostrate	broadly rounded, not protracted	broadly rounded, not protracted	broadly rounded, not protracted	acutely rounded, not protracted	broadly rounded, not protracted	broadly rounded, not protracted	obtusely rounded, never protracted
Helictoglossa	indistinct	absent	indistinct	absent	indistinct	distinct	indistinct	indistinct
Striation	transapical and parallel	absent	transapical and parallel	absent	transapical and parallel	transapical and parallel	uniseriate	transapical and parallel
Areolae in mantel	punctate	punctate	slit-like	slit-like	slit-like	slit-like	slit-like	slit-like
Number of dorsal striae (in 10 μm)	14–22	-	10–20	35–40	30–35	40–50	40–50	26–32
Number of ventral striae (in 10 μm)	20–36	70–80	22–30	40–50	30–45	40–55	-	36–42
Cingulum	open and perforated	unknown	open and plain	open and plain	unknown	open and plain	open and plain	unknown

*average (LM+SEM).

Ultrastructural observations using an SEM revealed distinct differences between the apices of the two species. *Paracatenulaporostriata* exhibited apical pores that were conspicuously absent in *C.javanica* (Fig. 2O). A notable difference was observed in the transapical striae, with *P.porostriata* having fewer (14–22/10 µm) compared to *C.javanica*, which had 26–32/10 µm (Table 2). Furthermore, the raphe slit of *P.porostriata* was significantly longer, measuring 11.8 µm, in contrast to *C.javanica*, which measured only 6.8 µm. The cingulum of *P.porostriata* was characteried by biseriate striae externally and a single row of elongated pores internally. However, both shared a similarity in biarcuate shape of the internal raphe branches.

### ﻿Novel *Wallaceago* species

Based on observations of ultrastructures by using an SEM, *Wallaceagoporostriatus* is characterised by an isosceles (semi-rhombic dorsiventral) triangular valve shape and a rectangular girdle shape. The established genus with a similar valve shape is *Seminavis* D.G. Mann, e.g., *S.basilica*. This species has a rhombic-lanceolate structure with truncated apices (Danielidis and Mann 2003). Distinct differences in the raphe structure differentiate the species *S.basilica* from *W.porostriatus*. Specifically, *S.basilica* is characterised by a straight raphe with a raphe fissure oriented towards the dorsal side. In contrast, *W.porostriatus* lacks such a raphe fissure. The raphe slit of *W.porostriatus* is notably short, measuring only 4.7 µm (Table 2). This is significantly shorter when compared to the similar genus *Catenulopsis*, which has a raphe slit length of 8.3 µm (Table 3; Kryk et al. 2021). Interestingly, the raphe slit length of *W.porostriatus* is nearly identical to that of *Medlinella*, which measures approximately 4.0 µm (Frankovich et al. 2016). The distal ends of the raphe in *S.basilica* and *Medlinella* exhibit a similarity to those of *W.porostriatus*, specifically, that they are curved towards the ventral side. However, the raphe of *W.porostriatus* is slightly arcuate in the middle, distinguishing it from the others. In general, raphe shape for *W.porostriatus* has slight curvatures whereas similar genera have raphe shapes that are more pronounced in bending or sinusoidal patterns along the ventral side. Another unique shape of the *W.porostriatus* raphe is attributed to the presence of a rabbet.

**Table 3.** Morphological characteristics of *Catenulopsis* species.

	*Ca.catenulafalsa*	*Ca.baweana* sp. nov.
	Kryk et al. (2021)	This study
Length (in 10 μm)	8.4–14.6	8.9–17.1
Width (in 10 μm)	3–4.1	1.7–3.1
Valve shape	dorsiventral	semi-lanceolate dorsiventral
Girdle shape	rectangular	rectangular
Sternum	narrow	narrow
Raphe slits path	biarcuate	biarcuate
Distal raphe ending	deflected to ventral (external), straight (internal)	deflected to ventral (external), straight (internal)
Proximal raphe ending	deflected to ventral (external), straight (internal), close	deflected to ventral (external), straight (internal), close
Raphe slits length (μm)	8.3	9.6
Raphe position	ventral area, very close to ventral margin	ventral area
Central nodule	absent	absent
Central area	present	present
Apices	obtusely rounded, never protracted	obtusely rounded, never protracted
Helictoglossa	indistinct	distinct
Striation	transapical and parallel	transapical and parallel
Areolae in mantel	slit-like	slit-like
Number of dorsal striae (in 10 μm)	30–40	14–20
Number of ventral striae (in 10 μm)	30–40	22–34
Cingulum	open and perforated	open and perforated

Among those species that have striation on the mantle, the type of striation differs. *W.porostriatus* has punctate striation, which means the striations appear as tiny, point-like dots. On the other hand, other species have slit-like striations, where the striations appear as small, elongated slits. The number of striations in the ventral area of the new species is higher, 70–80 per 10 µm, compared to *M.amphoroidea* (Tables 2, 3; Frankovich et al. 2016). Under LM, *W.porostriatus* and *M.amphoroidea* are identified as small diatoms, each with distinct valve lengths. The valve length of *W.porostriatus* ranges from 5.2–8.6 µm, while *M.amphoroidea* exhibits a slightly larger span of 7–13 μm. In contrast, *Ca.catenulafalsa* presents a longer valve length, measuring between 8.4–14.6 μm. Notably, the transapical groove in both *M.amphoroidea* and *Ca.catenulafalsa* is clearly visible under LM, a feature that is conspicuously absent in *W.porostriatus*.

### ﻿Novel *Catenula* species

*Catenulaboyanensis* shows similarities and distinct morphological characteristics with *C.javanica* (Table 2). Its valve length and breadth are slightly less than *C.javanica* (Table 2). The valve shape of this species is semi-lanceolate dorsiventral, and it has a rectangular girdle shape. In *C.javanica*, the valve shape is strictly lanceolate with a broader dorsal side. The primary distinction lies in the external positioning of the raphe. In the case of *C.javanica*, it is situated nearly along the edge of the ventral valve face. Conversely, for *C.boyanensis*, the raphe is located on the valve face itself, significantly further from the margin. In *C.boyanensis* the path of the raphe slits is straight, with the distal raphe ending being straight externally but deflected to the dorsal side internally. The proximal raphe ending is straight and distant, both externally and internally. The length of the raphe slits is 9.1 µm, and they are positioned in the ventral area. On the other hand, the raphe distinctly exhibits an undulating pattern in *C.javanica* externally and shares a similar pattern internally. The raphe slit length is lower at 9.1 µm. The number of dorsal and ventral striae for *C.boyanensis* is lower compared to *C.javanica* (Table 2). Upon observation with LM, the valve shapes of both species appear to be similar.

The diatom species *Catenuladecusa* shares similarities to *C.adhaerens* in terms of valve morphology and girdle structure. Both species exhibit a semi-lanceolate dorsiventral valve shape and a rectangular girdle form. However, notable distinctions can be observed between the two taxa. *C.decusa* is characterised by a shorter and slimmer frustule, with valves displaying a more pronounced lanceolate outline and a slender profile, in contrast to the broader, semi-lanceolate valves of *C.adhaerens* (length 6.8–12.4 μm, width 1.0–1.7 μm). Furthermore, the striation density is remarkably higher in *C.decusa*, with 35–40 striations per 10 μm, compared to the lower density of 25–35 striations per 10 μm observed in *C.adhaerens*. Additionally, the proximal ends of the raphe system in *C.decusa* exhibit a closer proximity 0.9 μm, while the distal ends are positioned nearer to the apices (0.8 μm). Another distinct difference is the thickening of the central area externally and internally in *C.decusa* with interrupted striation in the mantle, a feature absent in *C.adhaerens*. When observed under LM the central area seems lighter than other areas due to reflecting more light. Unlike *C.adhaerens*, *C.decusa* lacks helictogossae. In Kryk et al. (2021, p.10, figs 85, 93), the specimens that were incorrectly identified as *C.adhaerens* are *C.decusa*.

*Catenulakomodensis*. The newly described species is distinct and does not resemble previously described catenuloids. This diminutive diatom species has some valves shaped as linear rhombic dorsiventral and others mostly semi-lanceolate dorsiventral. The second difference is, internally, the presence of a second silica deposit close to the apices on the ventral side that splits the striation. This species shares some similarities with two other species, *W.porostriatus* and *Halamphoraveneta*, but *C.komodensis* is characterised by a smoothly arched dorsal margin, that distinguishes it from these related species. Valve morphometrics reveal a length ranging from 8.1–19.3 μm and a width spanning 1.1–2.1 μm, dimensions slightly larger than those of *W.porostriatus* (Table 2) yet approximately half the size of *H.veneta* (Levkov 2009). Notably, the proximal raphe endings are deflected towards the ventral side with a narrow gap between them, both externally and internally. The central nodule of *C.komodensis* extends towards the dorsal and ventral margins, manifesting itself as a thickening of the valve face similar to *C.decusa*. However, *C.komodensis* has two distinct silica deposits, situated in the central and apical areas, respectively. These deposits interrupt the striation patterns on both the dorsal and ventral aspects of the valve. Under LM, the valve shape is semi-lanceolate, and in smaller sizes, it appears lanceolate, and the silica thickening in the central and apical areas is clearly visible.

*Catenuladensestriata*. Newly described *C.densestriata* is similar to *Catenulaadhaerens*, *Catenulopsiscatenulafalsa*, and *Catenulopsisbaweana*. The significant difference is the number of dorsal mantle striae density in 10 μm which is 24–36 for *C.adhaerens* and 44–50 for the new species. Apical raphe ends are closer in proximity for *Catenuladensestriata* at 0.3–1.2 μm in contrast to 1.5 μm for *C.adhaerens*. Internally, raphe slits of *Catenulopsis* are straight versus *Catenuladensestriata* having raphe slits that are straight and apical raphe ends bent toward the dorsal side. One remarkable characteristic that distinguishes *C.densestriata* from other catenuloid species is its high striation density. Notably, the number of striations observed in this new species is approximately twice that of *C.adhaerens* (Sundbäck and Medlin 1986; Garcia and Talgatti 2011; Kryk et al. 2021), a significant characteristic of valve ornamentation.

Secondly, the distance between the external and internal proximal raphe endings in *C.densestriata* is nearly identical, measuring 1 μm and 0.9 μm, respectively. This feature is distinctly different from *C.adhaerens*, which exhibits twice the distance between its internal proximal endings and its external proximal endings (Kryk et al. 2021). Moreover, the distance between the distal raphe end and the apices is remarkably close in *C.densestriata*, 0.8 μm, with almost no difference between the external and internal morphologies. In contrast, *C.adhaerens* exhibits a greater distance of approximately 1.5 μm or twice that of the newly described species. Thirdly, the shape of the raphe slit’s path in *C.densestriata* exhibits an arcuate form, both internally and externally. Meanwhile, in *C.adhaerens*, the raphe slit is arcuate externally but straight internally (Kryk et al. 2021). Based on several distinguishing characteristics of *C.densestriata*, we believe that the specimen described as *C.adhaerens* in Kryk et al. (2021, p. 10, figs 84, 86) is *Catenuladensestriata*.

Based on the description and explanation provided, the four new species *C.boyanensis*, *C.decusa*, *C.komodensis*, and *C.densestriata* exhibit the following general characteristics: a dorsiventral valve shape, a rectangular appearance when viewed from the girdle perspective, and slit-like areolae present on both the dorsal and ventral mantles. Additionally, they possess an eccentrically positioned raphe on the ventral side. Notably, in the case of *C.boyanensis*, distinct transapical grooves are observed. Furthermore, these species exhibit an open and unperforated cingulum structure.

### ﻿Novel *Cantenulopsis* species

*Catenulopsisbaweana* sp. nov. (*Ca.baweana*) is the second species of the genus discovered and described thus far. The type of the genus is *Catenulopsiscatenulafalsa* (*Ca.catenulafalsa*) described from the tidal flat habitats in Nosy Be Island in NW Madagascar (Kryk et al. 2021). Whereas there is a distinct overlap in valve length between the two taxa, firstly, the valve width is smaller in the newly described species, with 1.7–3.1 μm versus 3–4.1 μm in the species from Nosy Be. The most distinct differences are: the girdle bands pore density, which is 30–40 in 10 μm in *Ca.catenulafalsa* and 50–65 in 10 μm in *Ca.baweana*; the striated valve in *Ca.catenulafalsa* versus the plain valve face in the newly described species; and strongly dorsally bent raphe slits in the former species versus slightly bent in the latter species. Furthermore, *Ca.baweana* has a semi-lanceolate shape with an abrupt transition between the valve face and mantle. In contrast, *Ca.catenulafalsa* has a smoother transition between these structures (see Kryk et al. 2021, figs 27–32). Secondly, the raphe of the new species is situated significantly distant from the valve margin. However, the raphe of *Ca.catenulafalsa* is situated in a transition area. Consequently, the distal raphe endings clearly extend into the mantle (Kryk et al. 2021). Thirdly, both species possess biarcuate raphe slits; however, in the new species, the raphe is straight in the central region and exhibits curvature at the proximal and distal ends. This contrasts with the raphe in *Ca.catenulafalsa*, which tends to follow a sinusoidal path and is shorter in length (Table 3). Fourthly, unlike *Ca.catenulafalsa*, which is characterized by the presence of ribs on the ventral valve face, the new species exhibits a distinct absence of such structures. Fifthly, the dorsal and ventral mantles of *Ca.catenulafalsa* are striated by the irregular shape of long solitary areolae, while the newly described species has a lower number in 10 µm of dorsal striae (Table 3).

### ﻿Key to catenuloid in this study with all described *Catenula* taxa

#### ﻿Order: Thalassiophysales

**Family: Catenulaceae**

**Key to genera**

**Table:** 

1	Cells dorsiventral, always solitary, shaped like the segment of an orange	***Amphora***
2	Cells dorsiventral, frequently from ribbon-like colonies, valves parallel to each other	***Catenula***
3	Cells dorsiventral tend to on rectangular, valves parallel to each other, raphe eccentric, distal and proximal ends curved to ventral	***Catenulopsis***
4	Cells dorsiventral, raphe biarcuate, eccentric distal ends curved to ventral	***Oxyamphora***
5	Cells dorsiventral, frequently from ribbon-like colonies, valves parallel to each other, present sulcus in apical area	***Paracatenula***
6	Cells dorsiventral, semi-rhombic, valve edge and raphe profiled rabbet, distal and proximal raphe ends curved to ventral	***Wallaceago***

#### ﻿Genus: *Catenula* Mereschkowsky

**Key to species**

**Table:** 

1	Valve shapes semi-lanceolate dorsiventral	**2**
1a	Valve shapes lanceolate dorsiventral	**9**
2	Valve length 6–20 μm	**3**
2a	Valve length 8–26 μm	**8**
3	Ventral margin straight	**4**
3a	Ventral margin curved	***C.brotasiae***
4	Internal valve has one thickening silica	***C.decusa***
5	Striae density 40–50 in 10 μm	***C.densestriata***
6	Striae density < 40 in 10 μm	***C.adhaerens***
6a	Transapical striae coarse	***C.robusta***
6b	Raphe eccentric along the edge of valve face	***C.javanica***
7	Striae density ~ 90 in 10 μm	***C.exigua***
8	Raphe eccentric on the ventral valve face	***C.boyanensis***
8a	Valve long 23–38 μm, wide 6–11 μm	***C.pelagica***
9	Internal valve has two thickening silica	***C.komodensis***

#### ﻿Genus: *Catenulopsis* Kryk, Witowski, Kociolek & Mayama

**Key to species**

**Table:** 

1	Valve dorsiventral semi-lanceolate, with sharp transition, raphe distant to valve margin, proximal and distal raphe ends bent to ventral, valve wide 1.7–3 µm, absent of ribs on ventral face, open and perforated cingulum	***Ca.baweana***
1a	Dorsiventral valve semi-lanceolate, smooth transition, raphe close to valve margin, proximal and distal raphe ends bent to ventral, valve wide > 3 µm, present of ribs on ventral face, open and perforated cingulum	***Ca.catenulafalsa***

#### ﻿Genus: *Paracatenula* Witkowski, Luthfi & M.Rybak, gen. nov.

**Key to species**

**Table:** 

1	Valve dorsiventral semi-lanceolate, with sharp transition, apices protracted rostrate, raphe straight, distal raphe ends bent to dorsal, striation transapical and parallel, 10.1–26.6 μm long, 1.7–4.7 μm wide, striae 20–36 in 10 μm, helictoglossa indistinct, open and perforated cingulum	***P.porostriata***

#### ﻿Genus: *Wallaceago* Witkowski, Arsad, Luthfi & M.Rybak, gen. nov.

**Key to species**

**Table:** 

1	Valve dorsiventral semi-rhombic, with sharp transition, raphe distant to valve margin, proximal and distal raphe ends bent to ventral, striation absent, 5.2–8.6 μm long, 0.9–1.2 μm wide, striae 70–80 in 10 μm, helictoglossa absent	***W.porostriatus***

## ﻿Conclusion

The present study significantly expands upon the foundational work of Kryk et al. (2021) by introducing 7 new catenuloids species from Indonesian marine environments. This discovery enriches our understanding of the biodiversity within the Catenulaceae family, particularly in the understudied regions of the Indonesian archipelago. Our ongoing research further supports the notion of a diverse diatom community within this archipelago, as evidenced by the identification of an additional amphoroid species that will soon be described as a new member of the Catenulaceae family. These findings collectively highlight the unexplored potential for discovering novel diatom taxa in the vast and ecologically complex coral reef ecosystems of Indonesia.

## ﻿Dedication

This article is dedicated to the memory of a researcher, diatomist, and our supervisor, the late Professor Andrzej Witkowski, who passed away on September 17, 2023. As a pioneering figure in the study of diatoms, his contributions to our understanding of these remarkable organisms have been invaluable.

## Supplementary Material

XML Treatment for Paracatenula

XML Treatment for Paracatenula porostriata

XML Treatment for Wallaceago

XML Treatment for Wallaceago porostriatus

XML Treatment for Catenula boyanensis

XML Treatment for Catenula decusa

XML Treatment for Catenula komodensis

XML Treatment for Catenula densestriata

XML Treatment for Catenulopsis baweana

## ﻿Additional information

### Author ORCIDs

Oktiyas Muzaky Luthfi https://orcid.org/0000-0002-9550-9381

Sulastri Arsad https://orcid.org/0000-0002-7322-7834

Adrian Kryk https://orcid.org/0000-0003-4959-4199

Yenny Risjani https://orcid.org/0000-0002-6191-5824

Yunianta https://orcid.org/0000-0002-9684-8131

Mateusz Rybak https://orcid.org/0000-0001-8998-9537

Łukasz Peszek https://orcid.org/0000-0002-9132-2210

Rafał J. Wróbel https://orcid.org/0000-0003-2593-0813

Janice L. Pappas https://orcid.org/0000-0002-1207-8388

Małgorzata Bąk https://orcid.org/0000-0001-6465-5047

Andrzej Witkowski https://orcid.org/0000-0003-1714-218X

### Data availability

All of the data that support the findings of this study are available in the main text.
